# High SARS-CoV-2 Seroprevalence after Second COVID-19 Wave (October 2020–April 2021), Democratic Republic of the Congo

**DOI:** 10.3201/eid2901.221009

**Published:** 2023-01

**Authors:** Yannick Munyeku-Bazitama, Gervais T. Folefack, Marc K. Yambayamba, Paul M. Tshiminyi, Benito M. Kazenza, John O. Otshudiema, Noe Tondri Guinko, Moreau D. Umba, Anastasie Mulumba, Lionel K. Baketana, Patrick K. Mukadi, Chris Smith, Jean-Jacques Muyembe-Tamfum, Steve Ahuka-Mundeke, Sheila Makiala-Mandanda

**Affiliations:** Hokkaido University, Sapporo, Japan (Y. Munyeku-Bazitama);; Université de Kinshasa, Kinshasa (Y. Munyeku-Bazitama, M.K. Yambayamba, B.M. Kazenza, P.K. Mukadi, J.-J. Muyembe-Tamfum, S. Ahuka-Mundeke, S. Makiala-Mandanda);; Institut National de Recherche Biomédicale, Kinshasa, Democratic Republic of the Congo (Y. Munyeku-Bazitama, P.M. Tshiminyi, L.K. Baketana, P.K. Mukadi, J.-J. Muyembe-Tamfum, S. Ahuka-Mundeke, S. Makiala-Mandanda);; Organisation Mondiale de la Santé, Kinshasa (G.T. Folefack, J.O. Otshudiema, N. Tondri Guinko, M.D. Umba, A. Mulumba); Nagasaki University, Japan (P.K. Mukadi, C. Smith);; London School of Hygiene and Tropical Medicine, London, UK (C. Smith)

**Keywords:** SARS-CoV-2, seroprevalence, second wave, Democratic Republic of the Congo, Kinshasa, COVID-19, coronavirus disease, severe acute respiratory syndrome coronavirus 2, viruses, respiratory infections, zoonoses, vaccine-preventable diseases

## Abstract

Serologic surveys are important tools for estimating the true burden of COVID-19 in a given population. After the first wave of SARS-CoV-2 infections, a household-based survey conducted in Kinshasa, Democratic Republic of the Congo, estimated >292 infections going undiagnosed for every laboratory-confirmed case. To ascertain the cumulative population exposure in Kinshasa after the second wave of COVID-19, we conducted a prospective population-based cross-sectional study using a highly sensitive and specific ELISA kit. The survey included 2,560 consenting persons from 585 households; 55% were female and 45% male. The overall population-weighted, test kit–adjusted SARS-CoV-2 seroprevalence was 76.5% (95% CI 74.5%–78.5%). The seroprevalence was 4-fold higher than during the first wave, and positivity was associated with age, household average monthly income, and level of education. Evidence generated from this population-based survey can inform COVID-19 response, especially vaccination campaign strategies in the context of vaccine shortages and hesitancy.

Two years after the first detected case of COVID-19 in Kinshasa, Democratic Republic of the Congo (DRC), the country experienced 4 subsequent waves of the virus, with peaks in June 2020 and January, June, and December 2021 (*1*). As observed across countries in Africa, the second wave in DRC was severe compared with the first wave in terms of disease incidence and associated deaths, partly because of lightening of stringent public health countermeasures implemented during the first wave, including international travel restrictions, and the spread of SARS-CoV-2 Beta variant (B.1.351) from southern Africa countries (*2*,*3*). By March 6, 2021, a total of 26,468 laboratory-confirmed cases were reported, including 712 virus-related deaths and 132,929 tests performed; Kinshasa accounted for nearly 75% of all reported cases (*1*).

The true burden of COVID-19 in Kinshasa is likely underestimated because PCR testing is conducted mainly on symptomatic persons meeting the case definition, omitting a large portion of persons who become infected with SARS-CoV-2 but are either asymptomatic or paucisymptomatic. Limited testing facilities throughout Africa, combined with the population’s underutilization of healthcare services, further widened the gap between the number of actual infections and detected cases (*2*,*4*). On the basis of data from a previously conducted household-based survey in Kinshasa after the first wave, we reported an infection-to-case ratio of 292:1 and a prevalence of 16.6% (*5*). The survey underscored the critical role of serologic surveys as complementary tools to routine testing results for guiding public health interventions.

Serologic surveys reveal the extent of infection within a given population and provide timely estimates on such key indicators as attack rate, mortality rate, and deaths, thus guiding public health actions and the development of evidence-based strategies (*6*). In the DRC particularly, natural infection immunity is more likely to outpace vaccine-induced immunization because vaccine rollout is hindered by factors such as vaccine hesitancy, low vaccine availability, and low vaccine coverage rates (*7*,*8*). Evidence is needed, therefore, to guide overall public health response, particularly vaccination strategies aimed at optimizing the use and delivery of available vaccines. We describe a population-based SARS-CoV-2 serosurvey conducted in Kinshasa after the second wave (October 2020–April 2021) of the COVID-19 epidemic to ascertain the cumulative population exposure.

## Methods

### Study Design and Population

We conducted a prospective population-based, cross-sectional study in Kinshasa on March 6–14, 2021, as part of the World Health Organization’s global framework for SARS-CoV-2 seroepidemiologic investigations (i.e., Unity Studies) (*9*). Kinshasa is the capital of the DRC and has an estimated population of 15 million, representing ≈15% of the population. Kinshasa is divided into 35 health districts, comprising 380 health areas.

**Figure 1:**
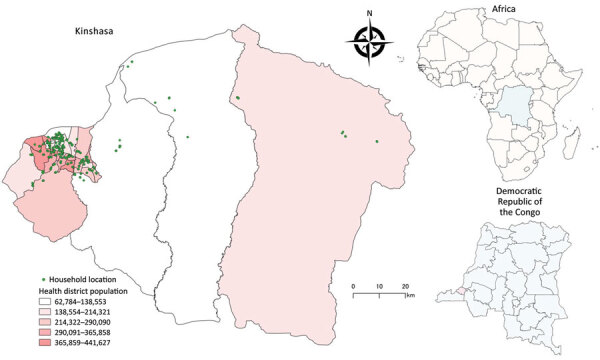
Study area for prospective, population-based, cross-sectional study to ascertain the cumulative population SARS-CoV-2 exposure in Kinshasa, Democratic Republic of the Congo, after the second wave of SARS-CoV-2. Inset maps show location of Kinshasa in Democratic Republic of the Congo (pink shading) and Democratic Republic of the Congo in Africa (blue shading).

We used a multistage, cluster sampling procedure to select study participants from a population spanning all 35 health districts of Kinshasa (Figure 1). We randomly selected 3 health areas within each health district by using probability sampling proportional to the size. After listing all streets or villages in each health area, we randomly selected 1 to 2 streets or villages within each area. We then listed all households in the selected streets or villages and systematically selected an average of 5 households from each health area. We determined eligible participants as persons of all ages who stayed in Kinshasa 2 weeks before the survey and had no contraindications to venipuncture. We obtained written informed consent from adults (participants ≥18 years of age) and emancipated minors, parental consent for participants <18 years, and assent for participants 10–17 years of age. The ethics committee of the Kinshasa School of Public Health reviewed the study (ESP/CE/81B/2020), and the study was aligned with the World Health Organization’s Unity Studies’ master protocol.

### Sample Size Calculation

We calculated the sample size based on the hypothesis of an expected seroprevalence of 20%, with a precision of 3%, a design effect of 2.2, and a nonresponse rate of 30%. We determined that >2,146 participants needed to be recruited.

### Data Collection

We presented a structured, pretested questionnaire to participants on an electronic tablet equipped with a mobile data-gathering application (Epicollect 5; Imperial College, London, https://www.imperial.ac.uk). The questionnaire covered questions regarding sociodemographic characteristics, medical history (with emphasis on COVID-19, hypertension [blood pressure ≥140/90 mm Hg], stroke, pulmonary disease, diabetes, chronic kidney disease, cancer, and obesity), alcohol and tobacco intake, SARS-CoV-2–related practices, and exposures to SARS-CoV-2. Exposures to SARS-CoV-2 comprised a previous SARS-CoV-2 infection, known contact with persons having suspected or laboratory-confirmed SARS-CoV-2 infection, and a history of travel to an affected province or country 2 weeks before the survey. We provided all participants with face masks and hand sanitizers and encouraged them to practice physical and social distancing.

### Blood Collection and SARS-CoV-2 Antibody Detection

We collected 3–5 mL of venous blood samples from eligible participants in red-topped plain tubes, which were transported at 4°C in cool boxes to the National Institute of Biomedical Research in Kinshasa the same day. At the institute, we processed blood specimens to obtain serum, aliquoted the serum in 2-mL cryotubes, and stored the tubes at –20°C for subsequent analyses.

We used the Wantai SARS-CoV-2 ELISA kit (Beijing Wantai Biologic Pharmacy Enterprise Co, Ltd, https://bjwtbp.en.ec21.com) to detect anti-spike IgG and IgM in a single replicate, according to the manufacturer’s instructions. We used known SARS-CoV-2–positive and SARS-CoV-2–negative samples as controls. We included prepandemic samples collected as part of measles surveillance, which tested negative for the measles virus serology (Appendix Figure 1). We considered a sample positive if the absorbance-to-cutoff ratio was >1.1. In the case of borderline results, we reran the test in duplicate and considered 2 matching results to be the final result.

The Wantai SARS-CoV-2 Total Antibodies ELISA kit has a sensitivity of 94.4% and a specificity of 100% (*10*). It detects whole antibodies against the receptor-binding domain (RBD) within the S1 subunit of the spike protein. The RBD represents approximately one third of the S1 subunit and is highly variable between SARS-CoV-2 and other betacoronaviruses (*11*). In this way, the Wantai SARS-CoV-2 Total Antibodies ELISA kit does not present cross-reactions with other coronaviruses that cause the common cold (i.e., OC43, HKU1, NL63, 229E). Besides providing high sensitivity and specificity, the Wantai kit offers dual detection of IgG and IgM, making the test kit useful in the very early phase of the disease course and in situations where the proportion of SARS-CoV-2 infections with asymptomatic or mild forms is prevalent; that is, when IgG synthesis is absent or low, and IgM is more likely to be abundantly synthesized and detected (*11*–*13*). The test kit also detects antibodies in most COVID-19 cases where the order of IgM-IgG seroconversion might not always be observed. In addition, antibodies directed against the RBD of the spike protein are strongly correlated with virus neutralization (*11*). The Wantai kit therefore can be helpful and informative as part of serologic surveys in gauging protective immunity in a general population.

### Statistical Analyses

We extracted data from the Epicollect 5 server, converted those results into a comma-separated values file, and transferred that information to Stata 15.1 (StataCorp LLC, https://www.stata.com) for analysis. We employed the svyset command to account for the survey design. We weighted estimates to reflect the population parameters. We used proportions with corresponding 95% CIs to summarize categorical variables and the mean or the median with standard deviation or interquartile range to summarize continuous variables. We used the Pearson χ^2^ test to assess the difference in seroprevalence between groups and the multivariable logistic regression to assess the association between SARS-CoV-2 seropositivity and key exposures. Finally, we corrected the seroprevalence to account for test kit performance as described elsewhere (*14*).

## Results

**Figure 2:**
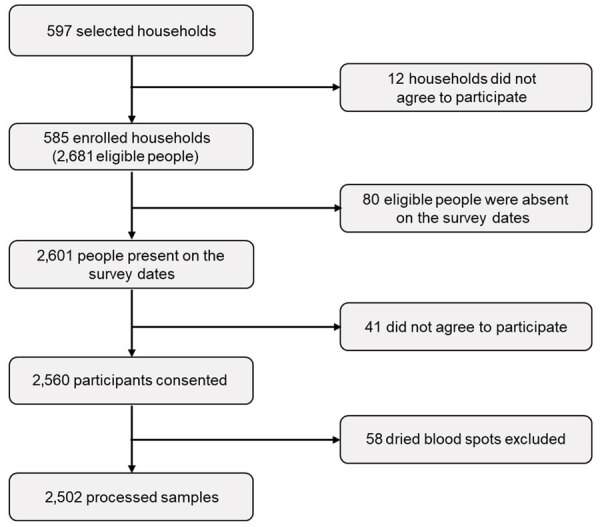
Flowchart of participants and household inclusion for prospective, population-based, cross-sectional study to ascertain the cumulative population SARS-CoV-2 exposure in Kinshasa, Democratic Republic of the Congo, after the second wave of SARS-CoV-2.

A total of 597 households were selected from 105 health areas (clusters), of which 585 (97.9%) agreed to participate in the survey. From the selected households, 2,681 persons were surveyed, of whom 2,601 (97%) were present on the survey dates. From the 2,601 eligible persons, only 2,560 (98.4%) consented to be interviewed and provided blood samples. Of the 2,560 blood samples, 58 dried blood spots were excluded. We successfully processed 2,502 (97.7%) samples, including 26 (1%) samples with borderline results that were not included in the analysis (Figure 2).

Of the 2,560 eligible participants, 1,412 (55.2%) were female and 1,148 (44.8%) were male. Most participants (1,787, 69.8%) were from health districts located in the western part of Kinshasa (Table 1). The median age was 30 years (interquartile range 18–46 years). Persons 20–29 years of age were the most represented (511/2,560, 19.9%), followed by those 30–39 years of age (409, 15.9%). The median size of the household was 7 (interquartile range of 6–9). Six households in 10 (349/585, 59.6%) reported an average monthly income of $51–$250 (US dollars), whereas nearly one quarter (145/585) reported an average monthly income of $1–$50. Most participants (1,209/2,560, 47.2%) had junior-high school education level, and 3.0% (77) had no formal education (Table 1).

**Table 1:** Sociodemographic and behavioral Characteristics of 2,560 study participants in a study to ascertain cumulative SARS-CoV-2 exposure in Kinshasa, Democratic Republic of the Congo, after the second COVID-19 wave*

Variables	Value
Sex	
F	1,412 (55.2)
M	1,148 (44.8)
Geographic area	
Western	1,787 (69.8)
Eastern	773 (30.2)
Median age, y (IQR)	30 (18–46)
Age group, y	
0–4	40 (1.6)
5–9	175 (6.8)
10–14	260 (10.2)
15–19	265 (10.4)
20–29	511 (19.9)
30–39	409 (15.9)
40–49	359 (14.0)
50–59	269 (10.5)
60–69	186 (7.3)
70–79	71 (2.8)
≥80	15 (0.6)
Household size, median (IQR)	7 (6–9)
Household average monthly income, US $	
1–50	634 (24.7)
51–250	1,525 (59.6)
251–500	340 (13.3)
501–1,000	56 (2.2)
>1,000	5 (0.2)
Education	
No formal education	77 (3.0)
Primary	473 (18.5)
Junior-high school	1,209 (47.2)
Secondary, vocational	268 (10.5)
Higher, vocational	46 (1.8)
University	487 (19.0)
Daily hand washing frequency	
<1 time	481 (18.8)
1–2 times	535 (20.9)
3–4 times	377 (14.7)
5–6 times	196 (7.7)
>6 times	971 (37.9)
Face mask wearing	
Never	422 (16.5)
Rarely	623 (24.3)
Sometimes	496 (19.4)
Often	659 (25.7)
Always	360 (14.1)
Alcohol intake	
N	1,721 (67.2)
Y	839 (32.8)
Tobacco intake	
N	2,410 (94.1)
Y	150 (5.9)

*Values are no. (%) except as indicated. IQR, interquartile range.

Regarding COVID-19 prevention measures, 37.9% (971/2,560) of participants reported washing their hands >6 times/day; another one fifth reported washing their hands daily (481 reported 1×/d, 535 reported 2×/d). More than one quarter of participants reported wearing a face mask frequently (659, 25.7%), but nearly one quarter reported rarely wearing a face mask (623, 24.3%). Most participants were nonsmokers (2,410, 94.1%) and 67.2% (1,721) reported no alcohol consumption (Table 1).

One in 2 participants reported >1 symptom indicative of COVID-19; fever was mentioned most frequently (713, 27.8%), followed by headache (627, 24.5%), chills (423, 16.5%), fatigue (409, 15.9%), and cough (400, 15.6%), (Table 2). Only 12.0% (308) of participants reported >1 comorbidity, with hypertension (166, 6.5%) and obesity (96, 3.7%) being the most reported. Most participants reported no contact with a laboratory-confirmed COVID-19 case (2,493, 97.4%) (Table 2).

**Table 2:** Clinical characteristics of 2,560 study participants in a study to ascertain cumulative SARS-CoV-2 exposure in Kinshasa, Democratic Republic of the Congo, after the second COVID-19 wave*

Variables	Value
Symptoms suggestive of COVID-19 2 weeks before survey	
N	1,280 (50.0)
Y	1,280 (50.0)
Fever	713 (27.8)
Headaches	627 (24.5)
Chills	423 (16.5)
Fatigue	409 (15.9)
Coughing	400 (15.6)
Runny nose	369 (14.4)
Myalgia	363 (14.2)
Abdominal pain	214 (8.4)
Sore throat	134 (5.2)
Nausea	132 (5.2)
Diarrhea	125 (4.9)
Anosmia/ageusia	83 (3.2)
Chest pain	73 (2.9)
Dyspnea	57 (2.2)
Median (IQR) no. symptoms	3 (1–4)
Comorbidity	
N	2,252 (88.0)
Y	308 (12.0)
Hypertension	
N	2,394 (93.5)
Y	166 (6.5)
Stroke	
N	2,540 (99.2)
Y	20 (0.8)
Asthma	
N	2,521 (98.5)
Y	39 (1.5)
Diabetes mellitus	
N	2,532 (98.9)
Y	28 (1.1)
Kidney injury	
N	2,553 (99.7)
Y	7 (0.3)
Cancer	
N	2,554 (99.8)
Y	6 (0.2)
Obesity	
N	2,464 (96.3)
Y	96 (3.7)
Pregnancy†	
N	1,371 (97.1)
Y	41 (2.9)
Contact with a laboratory-confirmed case	
N	2,493 (97.4)
Y	67 (2.6)

*Values are no. (%) except as indicated. IQR interquartile range. †Only women were considered for pregnancy.

The overall population-weighted SARS-CoV-2 seroprevalence was 72.2% (95% CI 69.8–4.4%) (Appendix Table 2). The seroprevalence was slightly higher, although not significantly, among female than male participants (73.8% vs. 70.1%; p = 0.146), and significantly higher in the western health districts of Kinshasa than in the eastern (74.3% vs. 68.3%; p = 0.021) (Appendix Table 1). Two health districts on either side of Kinshasa had the highest seroprevalence: Barumbu (88.4%, 95% CI 74.9%–95.1%) and Masina 2 (88.6%, 95% CI 77.1%–94.8%) (Appendix Table 2). Similarly, higher seroprevalence was found among participants 40–49 years of age (78.6%, 95% CI 72.9%–83.3%), those with university education (84.0%, 95% CI 69.9%–92.2%), and those who declared washing hands 5–6 times a day (76.8%, 95% CI 71.9%–81.0%) (Appendix Table 1). Most households (94.2%, 551/585) had >1 seropositive member; median was 3 positive members (Appendix Table 3, Figures 2, 3). After adjusting for the laboratory test kit performance, the overall seroprevalence increased from 72.2% (95% CI 69.8%–74.4%) to 76.5% (95% CI 74.5%–78.5%).

Participants living in households with an average monthly income of $51–$250 had 42% increased odds of SARS-CoV-2 infection compared with participants from a household with an average monthly income of $1–$50 (crude OR 1.42, 95% CI 1.12–1.80) (Table 3). In contrast, participants from households with an average monthly income of >$1,000 were 88% less likely to be infected with SARS-CoV-2 (crude OR 0.12, 95% CI 0.04–0.36). The likelihood of SARS-CoV-2 infection tended to increase with increasing education level and age. Participants with university-level education were >3 times more likely to be infected with SARS-CoV-2 than those without formal education (crude OR 3.73, 95% CI 1.10–12.67) (Table 3).

**Table 3:** Sociodemographic and behavioral characteristics associated with SARS-CoV-2 infection in a study to ascertain cumulative SARS-CoV-2 exposure in Kinshasa, Democratic Republic of the Congo, after the second COVID-19 wave*

Characteristic	Total no.	Seropositive, no. (%)	Crude OR (95% CI)	Adjusted OR (95% CI)
Sex				
F	1,369	1,005 (73.8)	Referent	Referent	
M	1,107	780 (70.1)	0.83 (0.64–1.07)	0.83 (0.62–1.06)	
Geographic area					
Western	1,724	1,291 (74.3)	Referent	Referent	
Eastern	752	494 (68.3)	0.74 (0.58–0.95)	0.81 (0.61–1.07)	
Age, y					
0–4	32	22 (70.8)	Referent	Referent	
5–9	165	98 (60.2)	0.62 (0.29–1.33)	0.38 (0.15–0.96)	
10–14	255	163 (61.8)	0.66 (0.28–1.55)	0.33 (0.12–0.91)	
15–19	258	187 (72.4)	1.08 (0.51–2.27)	0.40 (0.15 –1.06)	
20–29	497	364 (72.7)	1.10 (0.53–2.26)	0.39 (0.14–1.04)	
30–39	393	295 (74.8)	1.22 (0.57–2.62)	0.42 (0.15–1.18)	
40–49	349	272 (78.6)	1.51 (0.71–3.19)	0.52 (0.19–1.44)	
50–59	265	194 (74.0)	1.17 (0.53–2.63)	0.40 (0.15–1.08)	
60–69	179	131 (73.4)	1.14 (0.54–2.39)	0.41 (0.16–1.03)	
70–79	69	51 (76.6)	1.35 (0.51–3.56)	0.63 (0.24–1.68)	
≥80	14	8 (65.5)	0.78 (0.23–2.63)	0.39 (0.09–1.61)	
Household average monthly income, US $					
1–50	602	386 (66.8)	Referent	Referent	
51–250	1,471	1,099 (74.2)	1.42 (1.12–1.80)	1.22 (0.94–1.59)	
251–500	335	259 (75.3)	1.50 (0.98–2.33)	1.16 (0.73–1.82)	
501–1,000	53	35 (65.2)	0.93 (0.46–1.85)	0.81 (0.41–1.57)	
>1,000	5	1 (20.0)	0.12 (0.04–0.36)	0.12 (0.04–0.33)	
Education					
No formal education	66	41 (58.4)	Referent	Referent	
Primary	457	281 (62.2)	1.17 (0.56–2.44)	1.73 (0.72–4.17)	
Junior-high school	396	273 (69.7)	1.63 (0.82–3.24)	2.15 (0.88–5.23)	
High school	782	595 (74.6)	2.09 (1.04–4.17)	2.44 (1.02–5.86)	
Secondary, (vocational	263	203 (78.6)	2.61 (1.23–5.52)	3.02 (1.22–7.48)	
Higher, vocational	46	37 (76.2)	2.28 (1.07–4.83)	2.75 (1.06–7.14)	
University	466	355 (84.0)	3.73 (1.10–2.67)	4.33 (2.36–17.18)	
Daily hand washing frequency					
<1 time	466	297 (64.1)	Referent	Referent	
1–2 times	516	389 (75.4)	1.72 (1.18–2.50)	1.43 (0.89–2.31)	
3–4 times	362	248 (66.1)	1.09 (0.73–1.62)	0.91 (0.59–1.41)	
5–6 times	190	143 (76.8)	1.85 (1.30–2.64)	1.41 (0.85–2.36)	
>6 times	942	708 (74.8)	1.66 (1.20–2.31)	1.47 (0.92–2.36)	
Face mask wearing					
Never	398	247 (63.6)	Referent	Referent	
Rarely	611	440 (71.6)	1.43 (0.96–2.14)	1.14 (0.66–1.96)	
Sometimes	478	363 (74.8)	1.69 (1.19–2.41)	1.29 (0.80–2.07)	
Often	640	477 (74.5)	1.66 (1.15–2.39)	1.22 (0.73–2.03)	
Always	349	258 (74.1)	1.63 (0.96–2.76)	0.99 (0.51–1.96)	
*OR, odds ratio.					

On multivariable analysis, after adjusting for sex, age, and geographic area, an association emerged between SARS-CoV-2 infection and the 5–9-year age group (adjusted OR 0.38, 95% CI 0.15–0.96) and the 10–14-year age group (adjusted OR 0.33, 95% CI 0.12–0.91) (Table 3). SARS-CoV-2 infection remained associated with average household monthly income, especially for a household earning >$1,000 (adjusted OR 0.12, 95% CI 0.04–0.33). The association with education level remained and became stronger in effect size and statistical significance, especially for participants with university-level education (adjusted OR 4.33, 95% CI 2.36–17.18) (Table 3).

## Discussion

We conducted this population-based serologic survey at the end of the second wave (October 2020–April 2021) of COVID-19 in Kinshasa, DRC, before vaccination became available. As such, our results provide evidence of the cumulative exposure to SARS-CoV-2 among this population.

Our results show that, 1 year after detecting the first COVID-19 case, >3 out of 4 persons (76.5%) had been infected with SARS-CoV-2. This high seroprevalence indicates sustained community transmission in Kinshasa. Considering the Kinshasa population of 15 million in March 2021 (*15*), we estimate 11.5 million SARS-CoV-2 infections had occurred by March 6, 2021, but only 19,831 confirmed cases were reported (1 PCR-confirmed case for nearly 580 estimated infections). During the same period, 3,325 COVID-19 cases were active, and <100 hospitalizations occurred in COVID-19 treatment centers. Factors such as the younger age of the population, the predominance of mild and asymptomatic cases self-managed in the community, poor testing capacities, and low healthcare utilization might explain the discrepancies between reported cases, the actual number of infections, and the number of moderately or severely ill hospitalized persons.

The population-weighted and test kit–adjusted seroprevalence of 76.5% was nearly 4 times higher than that reported after the first wave (16.6%), reflecting an extensive community transmission after the lightening of lockdown measures, including the travel ban implemented during the first wave (*5*). Lower seroprevalence estimates were reported in Bangladesh (63.1%), Mali (58.5%), India (54.2%), Zimbabwe (53.0%), Kenya (44.2%), and Sierra Leone (2.6%) during similar periods (*16*–*22*). The characteristics of the test kit used and the variability in exposure levels across countries might explain differences in seroprevalence estimates. In our study and that of Bangladesh, an ELISA-based test detecting total antibodies against the RBD of the spike protein was used (*10*,*16*). Studies from Zimbabwe and India used serologic assays, targeting IgGs directed against the nucleocapsid protein, which are known to wane faster over time than those directed against the spike protein (*18*,*19*,*23*). Studies from Mali and Kenya used serologic tests that only targeted anti-spike IgG, thus missing newly infected persons who could bear anti-spike IgM rather than IgG (*17*,*20*). The Sierra Leone study used a lateral flow assay that targets both anti-spike IgM and IgG but is less sensitive than ELISA (*22*). Female participants were nearly 20% more likely to be infected than male participants, but the difference was not statistically significant. Similar results have been reported from other sub-Saharan Africa countries (*24*,*25*). As reported after the first wave of the COVID-19 epidemic in the DRC, the seroprevalence was not statistically different between western and eastern health districts of Kinshasa on multivariable analysis, although more cases were reported in western health districts (*5*). In our study, we observed a trend of increasing seroprevalence with age. This trend is consistent with other reports from Africa and Asia, which found higher exposures among participants 39–59 years of age (*16*,*17*,*20*,*22*). In addition, our results suggest that the second wave was characterized by similar infection rates for all age groups (*5*).

The risk for SARS-CoV-2 infection increased with average monthly household income up to $500 before decreasing dramatically, especially among households with incomes of >$1,000. Household income was associated with SARS-CoV-2 infection, and higher incomes reduced the risk for infection in households (*26*). The discrepancy in our study can be explained by respondent bias, because household income was assessed based on household heads’ responses rather than owned assets. Participants with a university education were more likely to be infected (84%), and having a university-level education was associated with a 4-fold increase in the risk for infection. A study from Portugal reported that a lower level of education was a critical risk factor for SARS-CoV-2 transmission compared with tertiary education (*27*). Higher levels of education are usually associated with better job opportunities and higher income and, thus, better living conditions and compliance with individual and collective protective measures. Conversely, a higher level of education and better employment may be associated with higher mobility and complex interactions with potentially infected persons, increasing the odds of infection.

Our study has several strengths, such as the robust sampling frame, which provided a large and representative sample size that included all 35 health districts of Kinshasa, and the high response rate among households (97.9%) and participants (98.4%). However, we were unable to perform a neutralizing antibody test on positive samples to ascertain protective immunity. In addition, there might have been respondent bias because we relied on self-reporting for variables such as comorbidities, household income, face mask wearing, daily hand washing, alcohol intake, and tobacco use. The stigma associated with COVID-19 might have played a role in underreporting critical information, as exemplified by the lower proportion (2.6%) of participants who reported a known contact with a laboratory-confirmed case. Finally, we collected clinical symptoms by interviewing participants, and there might have been recall bias, especially for symptoms that occurred more than 2 weeks prior to the survey.

In conclusion, our data suggest an extensive transmission of SARS-CoV-2 during the second COVID-19 wave in Kinshasa, resulting in a higher seroprevalence. Evidence generated from this population-based survey is critical to adjusting the COVID-19 response and especially vaccination campaign strategies in the context of vaccine scarcity and hesitancy, when a large proportion of potential vaccinees have been naturally exposed to SARS-CoV-2. The emergence and global spread of SARS-CoV-2 variants of concern, with their potential to resist neutralizing antibodies developed after natural infection, and antibodies waning could hamper the putative protective immunity. Serosurveillance coupled with neutralization tests and genomic surveillance of SARS-CoV-2 variants is needed to adjust the COVID-19 response plan in the DRC, including vaccination strategies, as the pandemic evolves.

Appendix Additional information for study of high SARS-CoV-2 seroprevalence after second COVID-19 wave (October 2020–April 2021), Democratic Republic of the Congo.
