# Supplementary material for: High SARS-CoV-2 Seroprevalence after Second COVID-19 Wave (October 2020–April 2021), Democratic Republic of the Congo
Source: Emerg Infect Dis. 2023 Jan;29(1):89–97. doi: 10.3201/eid2901.221009 (PMC9796206; doi:10.3201/eid2901.221009)
Supplement: Appendix — Additional information for study of high SARS-CoV-2 seroprevalence after second COVID-19 wave (October 2020–April 2021), Democratic Republic of the Congo. [file 22-1009-Techapp-s1.pdf]

Article DOI: http://doi.org/10.3201/eid2901.221009

High SARS-CoV-2 Seroprevalence after Second COVID-19 Wave (October 2020–April 2021), Democratic Republic of the Congo

Appendix

Appendix Table 1 SARS-CoV-2 seroprevalence by sociodemographic and behavioral characteristics (unadjusted estimates)

		Seropositive		Seronegative		
Characteristic	N	n (%)	95% CI	n (%)	95% CI	p
All study population	2,476	1,785 (72.2)	69.8–74.4	691 (27.8)	25.6–30.1	
Sex						
F	1,369	1,005 (73.8)	70.3–77.0	364 (26.2)	22.9–29.7	0.146
M	1,107	780 (70.1)	66.6–73.4	327 (29.9)	26.6–33.4	
Geographic area						
Western	1,724	1,291 (74.3)	71.9–76.5	433 (25.7)	23.5–28.0	0.021
Eastern	752	494 (68.3)	63.4–72.9	258 (31.7)	27.1–36.6	
Age group, y						
0–4	32	22 (70.8)	54.3–83.2	10 (29.2)	16.8–45.7	0.003
5–9	165	98 (60.2)	51.7–68.2	67 (39.8)	31.8–48.3	
10–14	255	163 (61.8)	53.4–69.6	92 (38.2)	30.4–46.6	
15–19	258	187 (72.4)	65.1–78.6	71 (27.6)	21.4–34.9	
20–29	497	364 (72.7)	68.0–77.0	133 (27.3)	23.0–32.0	
30–39	393	295 (74.8)	69.4–79.6	98 (25.2)	20.4–30.6	
40–49	349	272 (78.6)	72.9–83.3	77 (21.4)	16.7–27.1	
50–59	265	194 (74.0)	67.5–76.7	71 (26.0)	20.4–32.5	
60–69	179	131 (73.4)	66.0–79.8	48 (26.6)	20.2–34.1	
70–79	69	51 (76.6)	62.5–86.5	18 (23.4)	13.5–37.5	
≥80	14	8 (65.5)	39.9–84.5	6 (34.5)	15.5–60.1	
Household average monthly income (US $)						
1–50	602	386 (66.8)	62.6–70.9	216 (33.2)	29.1–37.5	0.003
51–250	1,471	1,099 (74.2)	71.3–76.9	372 (25.8)	23.1–28.7	
251–500	335	259 (75.3)	67.5–81.7	76 (24.7)	18.3–32.5	
501–1000	53	35 (65.2)	49.3–78.4	18 (34.8)	21.6–50.7	
>1000	5	1 (20.0)	8.0–41.7	4 (80.0)	58.3–92.0	
Education						
No formal education	66	41 (58.4)	41.4–73.6	25 (41.6)	26.4–58.6	<0.001
Primary	457	281 (62.2)	56.5–67.6	176 (37.8)	32.4–43.5	
Junior-high school	396	273 (69.7)	63.8–75.0	123 (30.3)	25.0–36.3	
High school	782	595 (74.6)	70.5–78.3	187 (25.4)	21.7–29.6	
Secondary (vocational)	263	203 (78.6)	73.6–82.8	60 (21.4)	17.2–26.4	
Higher (vocational)	46	37 (76.2)	70.3–81.3	9 (23.8)	18.7–29.7	
University	466	355 (84.0)	69.9–92.2	111 (16.0)	77.9–30.1	
Daily handwashing frequency						
<1 time	466	297 (64.1)	57.8–69.9	169 (35.9)	30.1–42.3	0.001
1–2 times	516	389 (75.4)	70.2–80.0	127 (24.6)	20.0–29.9	
3–4 times	362	248 (66.1)	59.9–71.7	114 (33.9)	28.3–40.0	
5–6 times	190	143 (76.8)	71.9–81.0	47 (23.2)	19.0–28.1	
>6 times	942	708 (74.8)	70.7–78.6	234 (25.2)	21.4–29.3	
Face mask-wearing						
Never	398	247 (63.6)	56.2–70.5	151 (36.4)	29.6–43.8	0.080
Rarely	611	440 (71.6)	67.4–75.4	171 (28.4)	24.6–32.6	
Sometimes	478	363 (74.8)	69.9–79.1	115 (25.2)	20.9–30.0	
Often	640	477 (74.5)	70.4–78.1	163 (25.5)	21.9–29.6	
Always	349	258 (74.1)	64.8–81.6	91 (25.9)	18.4–35.2	
Alcohol intake						

Characteristic	N	Seropositive n (%)	Seropositive 95% CI	Seronegative n (%)	Seronegative 95% CI	p
N	1,663	1,186 (71.6)	68.6–74.5	477 (28.4)	25.6–31.4	0.530
Y	813	599 (73.2)	69.2–76.8	214 (26.8)	23.2–30.8	
Tobacco Intake						
N	2,332	1,683 (72.3)	70.0–74.5	649 (27.7)	25.5–30.0	0.770
Y	144	102 (70.4)	56.3–81.5	42 (29.6)	18.5–43.7	

Appendix Table 2. SARS-CoV-2 seroprevalence by health districts in a prospective, population-based, cross-sectional study to ascertain the cumulative population exposure in Kinshasa, DRC, after the second wave of SARS-CoV-2.

Health districts	N	Seropositive n (%)	Seropositive 95% CI
All Kinshasa districts	2,476	1,785 (72.2)	69.8–74.4
Bandalungwa	72	56 (78.0)	69.8–84.6
Barumbu	76	66 (88.4)	74.9–95.1
Binza Météo	73	58 (77.9)	57.2–90.3
Binza Ozone	65	41 (61.3)	47.5–73.5
Biyela	71	39 (52.6)	43.0–62.0
Bumbu	71	57 (75.3)	51.7–89.7
Gombe	66	51 (78.8)	67.5–86.9
Kalamu 1	75	55 (72.6)	65.8–78.5
Kalamu 2	71	41 (57.7)	48.7–66.1
Kasavubu	70	55 (79.3)	65.8–88.4
Kikimi	75	58 (78.1)	70.1–84.5
Kimbanseke	74	36 (49.5)	34.3–64.8
Kingabwa	81	53 (74.5)	59.5–85.3
Kingasani	73	57 (78.1)	70.0–84.4
Kinshasa	73	51 (70.3)	58.5–79.8
Kintambo	69	56 (83.6)	63.9–93.6
Kisenso	43	36 (81.9)	72.8–88.4
Kokolo	78	55 (69.3)	58.5–78.3
Lemba	70	51 (67.3)	50.7–80.5
Limete	70	47 (67.2)	58.5–74.8
Lingwala	72	61 (82.1)	72.9–88.5
Makala	69	43 (62.1)	54.7–69.0
Maluku 1	54	39 (70.5)	63.2–76.8
Maluku 2	65	28 (44.3)	35.9–52.9
Masina 1	70	44 (61.1)	45.8–74.5
Masina 2	75	66 (88.6)	77.1–94.8
Matete	76	65 (85.3)	75.5–91.6
Mont Ngafula 1	77	64 (84.0)	77.2–89.1
Mont Ngafula 2	70	49 (70.1)	62.9–76.4
Ndjili	82	61 (72.0)	44.2–89.3
Ngaba	63	46 (72.9)	63.1–81.0
Ngiri Ngiri	71	60 (84.8)	73.3–91.9
Nsele	69	41 (67.8)	54.1–78.9
Police	67	44 (67.9)	52.7–80.1
Selembao	80	55 (70.1)	63.0–76.3

Appendix Table 3. Distribution of seropositive households (N=585) from a prospective, population-based, cross-sectional study to ascertain the cumulative population exposure in Kinshasa, DRC, after the second wave of SARS-CoV-2.

Variable	n	%
Seropositive household		
N	34	5.8
Y	551	94.2
Seropositive members		
<2	89	16.2
2–3	257	46.6
4–5	146	26.5
>5	59	10.7

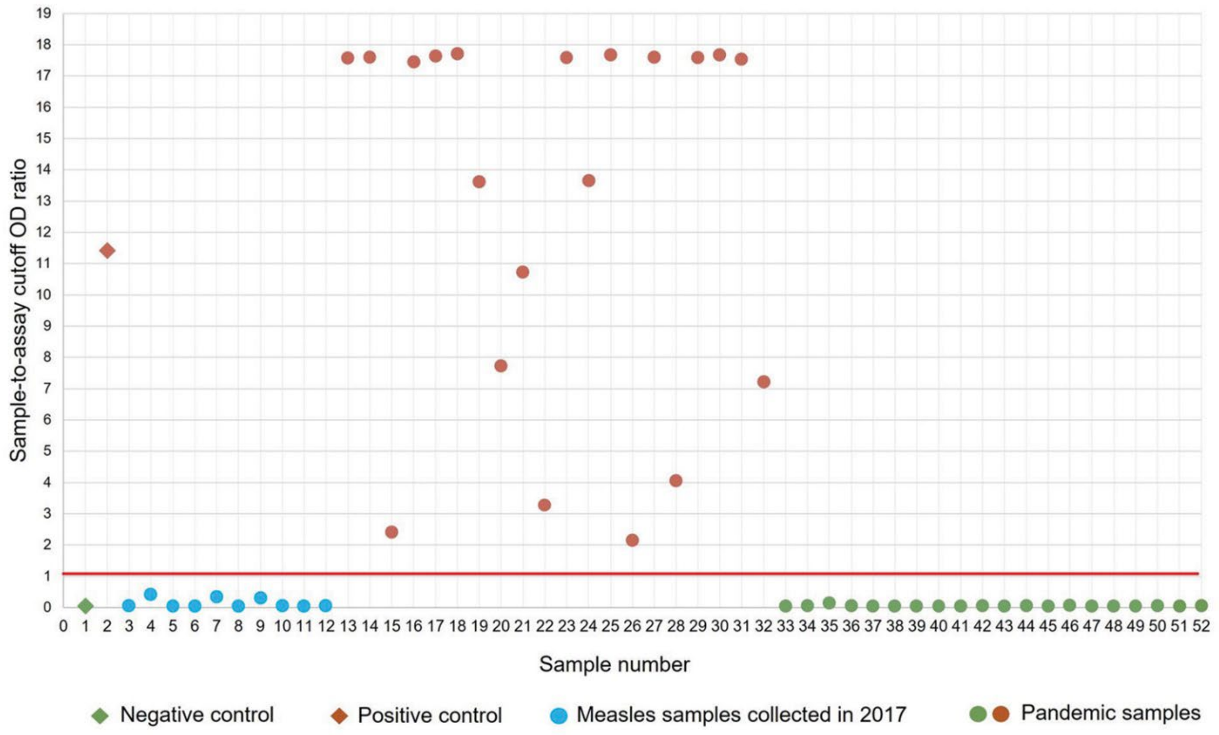

Appendix Figure 1. Pre-pandemic and pandemic samples screening with WANTAI SARS-CoV-2 Total Antibodies ELISA kit for a prospective, population-based, cross-sectional study to ascertain the cumulative population exposure in Kinshasa, DRC, after the second wave of SARS-CoV-2. Ten measles antibody-negative serum samples were randomly selected from samples collected in 2017 as part of measles surveillance. An additional 20 known positive and 20 known negative pandemic samples were concurrently tested. Sample to assay cutoff OD ratios are presented with a positivity threshold ratio of at least 1.1. The known SARS-CoV-2 antibody positive and negative samples were tested using a highly sensitive and specific Luminex-based assay detecting IgG directed against the nucleocapsid protein and the spike protein (Nkuba et al, https://www.ncbi.nlm.nih.gov/entrez/query.fcgi?cmd=Retrieve&db=PubMed&list_uids=34089598&dopt=Abstract).

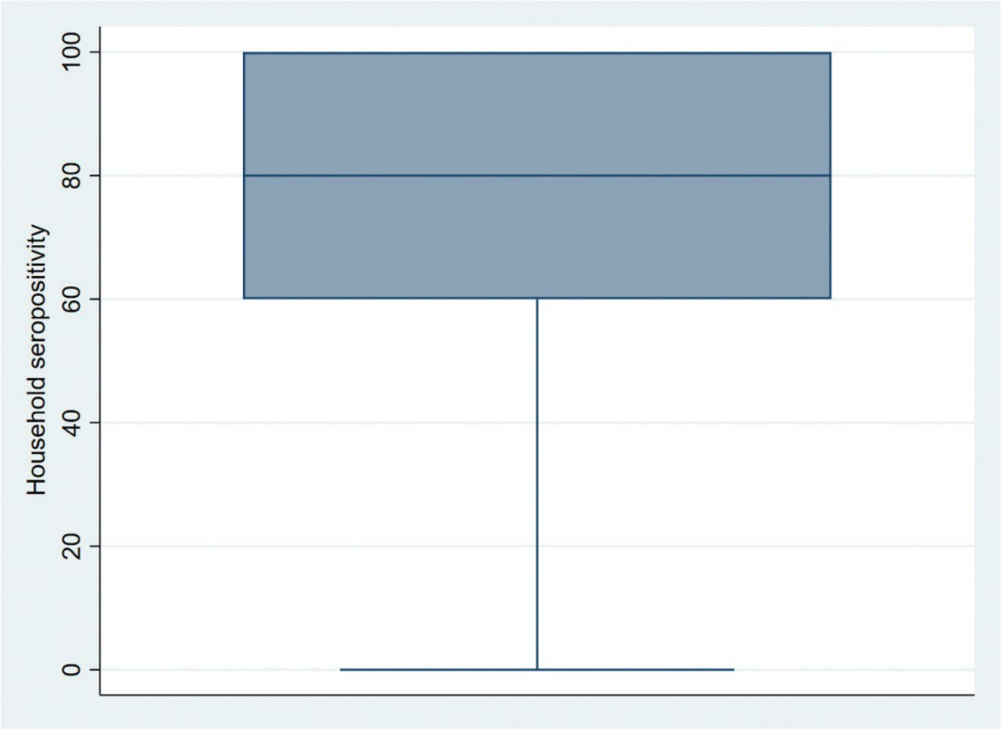

Appendix Figure 2. Distribution of seropositivity by household for a prospective, population-based, cross-sectional study to ascertain the cumulative population exposure in Kinshasa, DRC, after the second wave of SARS-CoV-2.

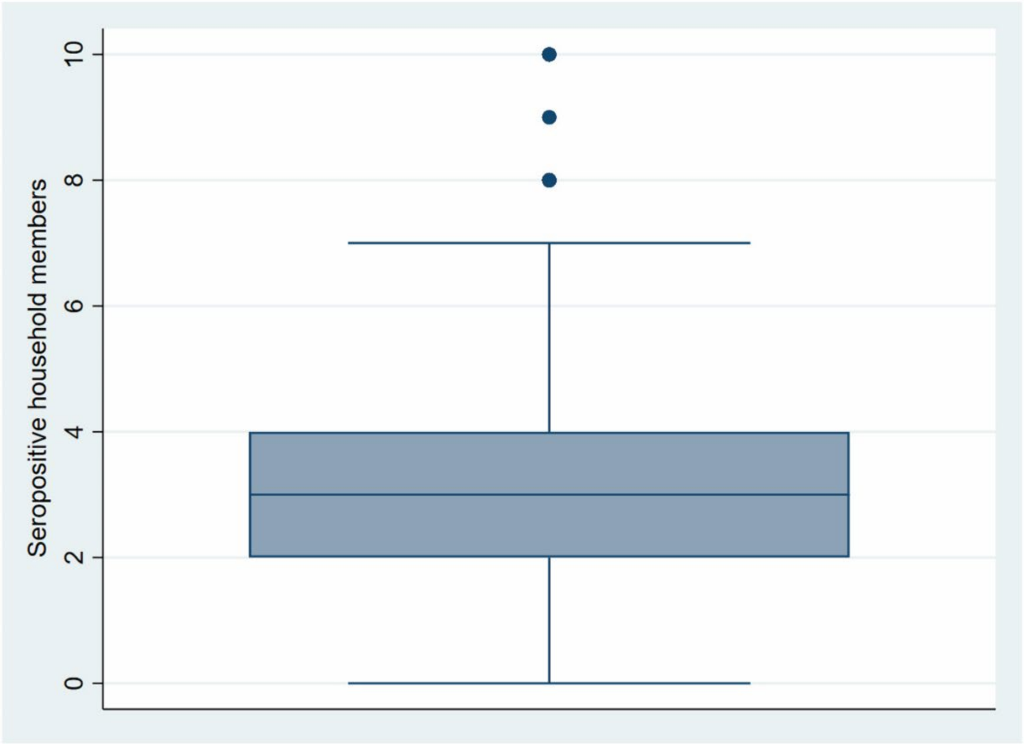

Appendix Figure 3. Distribution of seropositive household members in a prospective, population-based, cross-sectional study to ascertain the cumulative population exposure in Kinshasa, DRC, after the second wave of SARS-CoV-2.
